# Conversational pragmatics: memory reporting strategies in different social contexts

**DOI:** 10.3389/fpsyg.2023.1004524

**Published:** 2023-05-25

**Authors:** Beatriz Martín-Luengo, Karlos Luna, Yury Shtyrov

**Affiliations:** ^1^Centre for Cognition and Decision Making, Institute for Cognitive Neuroscience, National Research University Higher School of Economics, Moscow, Russia; ^2^Department of Psychology, Universidad Nacional de Colombia, Bogotá, Colombia; ^3^Center of Functionally Integrative Neuroscience (CFIN), Department of Clinical Medicine, Aarhus University, Aarhus, Denmark

**Keywords:** conversational pragmatics, memory reporting, confidence, social context, reporting strategies

## Abstract

Previous studies in conversational pragmatics have showed that the information people share with others heavily depends on the confidence they have in the correctness of a candidate answer. At the same time, different social contexts prompt different incentive structures, which set a higher or lower confidence criterion to determine which potential answer to report. In this study, we investigated how the different incentive structures of several types of social contexts and how different levels of knowledge affect the amount of information we are willing to share. Participants answered easy, intermediate, and difficult general-knowledge questions and decided whether they would report or withhold their selected answer in different social contexts: formal vs. informal, that could be either constrained (a context that promotes providing only responses we are certain about) or loose (with an incentive structure that maximizes providing any type of answer). Overall, our results confirmed that social contexts are associated with different incentive structures which affects memory reporting strategies. We also found that the difficulty of the questions is an important factor in conversational pragmatics. Our results highlight the relevance of studying different incentive structures of social contexts to understand the underlying processes of conversational pragmatics, and stress the importance of considering metamemory theories of memory reporting.

## Introduction

Efficient communication and exchange of information are essential for ensuring our survival in a dynamic environment. Yet, there are many issues in research on human communication and, more specifically, in conversational pragmatics that remain under-investigated. Most of the research on conversational pragmatics has been conducted from the point of view of what the *listener* would understand from the *speaker’s* intended meaning (Dieussaert et al., 2002; Papafragou and Musolino, 2003; De Neys and Schaeken, 2007; Mazzone, 2013; Cruz, 2014). For example, in a study focused on the influence of the content and context on the understanding of others’ reasoning, Dieussaert et al. (2002) found that one of the most imperative variables for the *listener* to understand conditional statements was the speaker’s control of how the information was delivered. Similarly, Huang and Snedeker (2009) studied the effect of the qualifier used by the speaker (some/all, less/much) on what the *listener* will understand. A recurrent result is that, e.g., from a sentence like “Some of the presents are ready” most listeners understand that “Not all presents are ready,” even though the use of the qualifier *some* is compatible with the qualifier *all*. However, this type of research in conversational pragmatics mainly covers one side of the coin in communication, that of the listener. To gain a more comprehensive understanding of communication processes in context, we also need to understand it from the *speaker’s* side.

In the present study, we report an experiment in which we studied the effect of the incentive structures subjacent to social situations in communication. To do so, we manipulated the type of social context where the communicational exchange takes place and the level of difficulty the speakers experience in providing the information in their message. We first review evidence about how people regulate the amount of information they provide in the absence of a particular social context, and relate this evidence to the current metamemory theories of memory reporting. We then review studies on conversational pragmatics focusing on the regulation of informativeness in different social contexts. After that, we briefly review how the difficulty of finding the to-be-retrieved information may influence the information reported in such studies and, finally, we present the current study and our hypotheses.

### Tailoring a message: the report option

People do not speak in the same way and use the same language if they are talking to a close friend, a work colleague, or a stranger. Research in conversational pragmatics from the speakers’ side confirm this observation: participants tailor the information they provide depending on the social context (Vandierendonck and Van Damme, 1988; Gibbs and Bryant, 2008; McCallum et al., 2016; Martín-Luengo et al., 2018). For example, participants provide different amount of detail when they retell a previously presented story depending on the key audience they were approaching (e.g., Vandierendock and Van Damme, 1988, who used peers, public contest, or “Martians” as audience).

This message tailoring is heavily influenced by the confidence in the correctness of the information to share, which is an integral element of the *metamemory* process. Metamemory research is a part of the larger metacognitive field devoted to the study of the subjective experience related to memory (Nelson and Narens, 1980). Metamemory is crucially important in many aspects of life; for instance, depending on this subjective feeling, students might decide to spend more time rehearsing the lessons in case they feel they would not achieve the desired grade (Higham and Arnold, 2007; Arnold et al., 2013), eyewitnesses might show themselves more certain about what they think they saw during a crime (Roberts and Higham, 2002), etc. There are different theoretical approaches that aim to explain the roots of metacognitive ratings: “direct access” or “trace-strength” (e.g., Hart, 1965; Dunlosky and Nelson, 1994), where the confidence ratings are meant to be based on the strength of memory retrieval; or the “cue-utilization” framework (Koriat, 1997) that states that people make confidence judgments based on the complementary information they access as well as their past performance on the particular task. Regardless of its underlying mechanism of metamemory judgments, what matters to the present research is that the confidence we have about the information we retrieve will affect the amount of information we share depending on the social context in which we are being questioned (McCallum et al., 2016; Martín-Luengo et al., 2018).

One simple and efficient way to tailor a message is to decide whether to report a given piece of information or withhold it, effectively modifying the message depending on the circumstances. This decision, termed *the report option* (Koriat and Goldsmith, 1996), is grounded in the metamemory processes of monitoring and control and is made based on a number of factors. The most relevant are (1) the *confidence* that the answer is correct and (2) the *informativeness* of the resulting answer (Goldsmith, 2016). In a conversational exchange, the speaker internally produces a piece of information, which is evaluated for its likelihood of being correct (confidence) and usefulness (informativeness). Subjective confidence and informativeness are then compared against previously self-defined minimum criteria for a given situation and, if both criteria are satisfied, then the answer is reported. Otherwise, it is withheld. Alternatively, if the criteria are not met, then the information can also be modified and a new comparison against criteria is made. There are several modifications that can be made depending on the nature of the information (see, for example, Luna and Martín-Luengo, 2017; see also Goldsmith, 2016, for a review), but here we will focus on the report option and the decision to report or withhold an answer.

Notably, there is an experimental measure that combines both report choices and their confidence, and thus allows objective quantitative study of the thresholds that individuals use to base their decisions on: the so-caleld Report-Criterion Probability (*P_rc_*). This measure, along with the respective experimental procedures, was introduced by Koriat and Goldsmith (1996; for further explanation on computation, see also Goldsmith and Koriat, 2007) and is based on raw confidence responses. Thus, the use of this procedure enables scrutinizing participant’s choices, which was one of the aims of this study.

Importantly, previous research has shown a trade-off between accuracy and informativeness in the information people provide (Ackerman and Goldsmith, 2008). One way to control this trade-off is the report option: in order to provide highly accurate information, people might in fact need to withhold some of the information, but, on the other hand, when they want to maximize informativeness, they would report more information even though it may increase the chances of providing an incorrect answer (Koriat and Goldsmith, 1996). Several factors affect these opposing tendencies to focus on informativeness and provide more elaborate answers as opposed to focussing on accuracy and reporting less elaborate/fewer answers. One of these factors is the *social context*.

### Social context and its incentives structure

The aforementioned informativeness and confidence criteria are affected by communication norms and pragmatic considerations relevant to a given context (Goldsmith, 2016). For example, Martín-Luengo et al. (2018) and Martín-Luengo et al. (2023)^1^ manipulated context formality and studied the tendency to report or withhold answers in formal and informal contexts. A context is formal when there are explicit rules that govern the situation, for example a job interview or a court testimony. A context is informal when such rules do not exist or are more flexible, for example when chatting with friends or being at a party. Past research has found that in formal settings participants are careful in balancing the information they report or withhold in order to increase the chances that the information they do report is in fact correct, whereas in informal settings participants tend to report almost all their possible answers regardless of their confidence in correctness (Martín-Luengo et al., 2018, see text footnote 1).

However, the metacognitive literature proposes that it is not the presence or absence of explicit rules that directly affect communication, but rather the incentive structure of a given context that prompts different reporting strategies (Goldsmith, 2016). The incentive structure of a context refers to the perceived penalties and rewards for providing accurate answers or for providing informative answers. For example, in some contexts it may be more important to provide accurate information, that is, the situation rewards accuracy (e.g., successfully passing an exam), but in other contexts it may be more important to provide rich information, that is, the situation rewards informativeness (e.g., being accepted as a socially apt person at an informal event). Thus, we can speculate that it is not the context *per se* which prompts different reporting strategies, but the association of the context with an underlying incentive structure which favors accuracy or informativeness. Depending on that incentive structure, people will employ different reporting strategies (Goldsmith, 2016). Also, it is important to mention that the perceived incentive structure of a given situation may vary from person to person and even within the same person between different moments of time. Therefore, in this research, we studied people’s perceptions and assumptions about the *expected* incentives in different social contexts.

As mentioned, previous research studied the reporting strategies in formal and informal contexts (Martín-Luengo et al., 2018, see text footnote 1). However, it does not seem very reasonable to assume that any formal or informal situation is associated with a specific incentive structure and that it would promote the same report pattern. In this research, we propose that the incentive structure may also vary within different formal and informal contexts. For example, some contexts are characterized by the social pressure to present ourselves in the best possible light and make the best impression regarding our skills or knowledge, such as at a job interview (a formal context). When the chances of providing an incorrect answer are high, admitting some ignorance and not providing any answer might be more socially acceptable (Ackerman and Goldsmith, 2008, Exp. 3). As mentioned above, in such formal contexts people might tend to report information only when they have high confidence in its accuracy. In other words, answers are constrained by confidence; thus, we termed these contexts as “constrained.” The incentive structure of constrained contexts would favor accuracy. In some other settings, there may be a social demand to provide information and a tendency to report everything. For example, when testifying in a trial (also a formal context) eyewitnesses are pressured to collaborate by giving all the information they might know about a criminal event regardless of how certain they feel about it (McCallum et al., 2016). In this type of context, there is no such confidence constraint, and thus the confidence in response correctness basically has no relevance. For example, Paulo et al. (2016) showed that during a police interview witnesses produced statements accompanied by markers of uncertainty (e.g., “*I believe*,” “*maybe*”), showing that they were willing to report information even though they had low confidence in it. Thus, reporting some information (i.e., the overall informativeness of the answer) is perceived as more relevant than the accuracy of the retrieved memory. In these contexts, the answering strategy is better described as “loose” and the incentive structure would favor informativeness, even though the overall context is still very formal.

A parallel case can be made for informal contexts. For instance, a “first date” situation is an example of an informal context likely prompting a constrained answering strategy, because there are no explicit rules and people probably want to only share what they are confident in. In turn, an informal context with a loose answering strategy may be the situation of chatting with friends. Based on these definitions, it is possible to draw some similarities between the incentive structure at a job interview and at a date despite one context being formal and the other informal. In both cases, people would not report a lot of information unless they are reasonably certain about its correctness, that is, they favor accuracy. Likewise, there are similarities between the incentives when testifying in a trial and chatting with friends. In both loose contexts the incentive would be to report maximum information, that is, they both favor informativeness, even though they differ in formality. To verify this suggestion, the present research tested the different patterns of reporting derived from the different incentive structure of each context.

### Difficulty of questions

Another issue, which has been treated differently in studies on conversational pragmatics, is the difficulty of the material. In some studies, participants had access to the information during the main task (Gibbs and Bryant, 2008) or the information was presented a few minutes beforehand (Vandierendonck and Van Damme, 1988), which made the tasks relatively easy. In other studies, difficult general-knowledge questions were used under the assumption that answers to easy questions would be reported regardless of the context (Martín-Luengo et al., 2018, see text footnote 1). This idea is consistent with the criteria to report a piece of information for which, for easy questions, confidence would probably be high enough to satisfy the confidence criterion, and thus participants will not have to withhold information. However, this idea was never empirically tested. Therefore, our secondary objective in this research was to examine the effect of incentives on the willingness to report or withhold information when answering questions with varying levels of difficulty.

### Present research

In the present experiment, we studied the effects of the incentive structure prompted by different social contexts. We manipulated both the formality of the context (formal, informal) and the answering strategy the context elicits (loose, constrained) in communicating individual responses to easy, intermediate, and difficult questions. We particularly focused on the decision to report or withhold information in a conversational exchange as a window onto the information the speaker wants to provide. In line with the previous research (Martín-Luengo et al., 2018, see text footnote 1) reviewed above, we expected that informal contexts would be associated with an incentive favoring informativeness and thus expected our participants to report more information in informal than in formal contexts. We also expected that loose contexts would be associated with informativeness and thus the participants would report more information in loose than in constrained contexts. Finally, if question difficulty is more relevant than social context incentives in the decision to report or withhold information, we should not find any effect of context on reporting in easy, intermediate, or difficult question conditions. Alternatively, if the context has any influence on memory reporting beyond question difficulty, we should observe different patterns of reporting/withholding across difficulty levels. If this is the case, then we would expect the effect of context mentioned above, but it would be limited to intermediate and difficult questions because for easy questions there may be no need to regulate the amount of information reported.

To test these hypotheses, we conducted an experiment in which participants answered general-knowledge questions and rated the confidence in their answers prior to being informed of the specific context. After that, one of the four possible social contexts was introduced and participants decided whether to report or withhold their answer in that specific context. We used a large amount of general-knowledge questions validated previously in a large participant sample (Martín-Luengo et al., 2020) to ensure that each context had a similar amount of easy, intermediate, and difficult questions in a counterbalanced fashion.

## Method

### Participants

Twenty-five participants (11 females, mean age = 21.03, *SD* = 3.92) recruited on social media took part in the experiment for a small monetary compensation. Most of the participants were in the process of completing their university studies and the rest had completed high-school education. All were native Russian speakers. It is worth to mention that in Russia it is compulsory to pass the Unified State Exam for obtaining the high-school diploma. This guarantees that all of our participants have a similar basic general-knowledge. The sample size was decided on the basis of previous research on the topic (Martín-Luengo et al., 2018; *n* = 24). Although such sample could in principle be considered limited with respect to the statistical power, the homogeneity of the sample (similar age, origin, and educational background) and the high number of items from different topics (see below) and varying levels of difficulty lead to smaller inter-participant variability than inter-trial variability. In that case, the large number of items used in the experiment increases power (Rouder and Haaf, 2018). Thus, we are reasonably certain that our study was powered enough as to the objectives of this research.

### Materials and design

Four hundred and two multiple-choice general-knowledge questions (GKQ) were used in the experiment. Four hundred questions were used in the main experiment and two were used for practice. The questions were selected from a previously normed and validated database of questions (Martín-Luengo et al., 2020). The GKQ covered different topics: history, chemistry, biology, literature, spelling and grammar, and geography. The GKQ were selected to include all levels of difficulty: easy, intermediate, and difficult. For each question, participants selected one out of four alternatives and rated how confident they were in their answers on a scale from 0% (totally unsure) to 100% (totally sure), graded in 10% steps. The design was a 2 formality (formal, informal) × 2 answering strategy (constrained, loose) × 3 question difficulty (easy, intermediate, difficult), with the three variables manipulated within participants in a fully counterbalanced fashion. Main measures were accuracy, confidence in the correctness of the selected answer, and proportion of reported answers.

### Procedure

The experiment was programmed using Experiment Builder 2.3.38 (SR Research, Toronto, Canada), and consisted of one practice session with two questions and eight experimental blocks with 50 questions each. The order of the questions in the experimental blocks, the placement of the alternatives on the screen, and the social context for each question were fully counterbalanced. No more than two questions were placed in the same context in a row. All participants had the same practice questions, which were not used in the main experiment.

**Figure 1:**
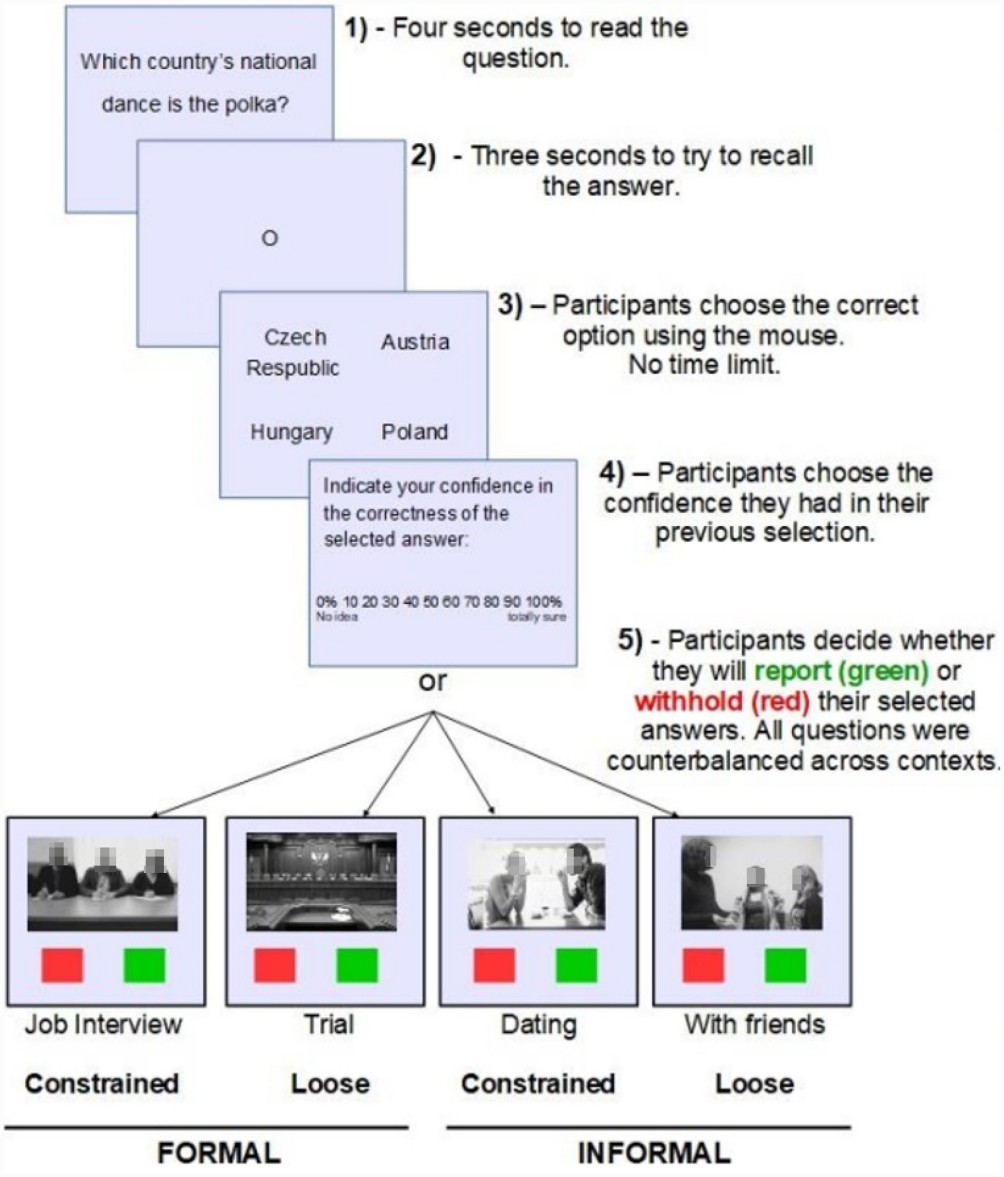
Experimental procedure for each trial.

Figure 1 shows an overview of the steps followed on a given trial and Table 1 shows the descriptions of the pictures for each context that appeared in Figure 1. Participants were tested individually using a personal computer. First, they read and signed the informed consent form and provided demographic data. Then, they read the instructions and completed two training questions (different from the main set of stimuli) before beginning the main experimental phase. A question appeared on the screen for 4 s and then participants were instructed to try to retrieve the answer from memory within 3 s. After that, participants were shown four alternative answers and had to use the mouse to click on the correct one. The participants then rated their confidence that their answer was correct. Finally, participants were shown a specific context which consisted of a picture with a brief description (see Table 1) and chose whether they preferred to report or withhold their answer in that specific context. The experiment lasted approximately 2.5 h. To prevent fatigue, participants were given the chance to take a brief rest after each question. There were also breaks of 2–3 min between blocks in which participants had the chance to leave the room, stretch their muscles, eat or drink something, and use the toilet if needed.

**Table 1:** Verbal descriptions of each of the four conditions given to participants (translated into English).

Formal contexts	Constrained	*Job Interview*: Imagine that you are in an important job interview. You really need this job. You feel the tension, but you still try to look like an expert in the field.
	Loose	*Court*: Imagine that you are testifying in court and that the judge is asking you questions. You do not know whether your answers will be helpful, but they might help to convict the killer.
Informal contexts	Constrained	*Dating*: Imagine that you are hanging out with someone you would like to date. You are having fun but you are still trying to make a good impression.
	Loose	*Conversation with friends*: Imagine that you are with friends, having a good time. You feel relaxed and glad to be with them. You are having a cheerful conversation about different topics.

## Result

We first report a summary of the results of the analyses of accuracy and confidence. Then, we present the analyses to test our hypotheses based on the proportion of reported answers and finally, *P_rc_* (Report-Criterion Probability) analyses (Koriat and Goldsmith, 1996).

Unless otherwise mentioned, we report 2 formality (formal, informal) × 2 answering strategy (constrained, loose) × 3 question difficulty (easy, intermediate, difficult) analysis of variance (ANOVA), and pairwise comparisons using the Student’s *t*-test with Bonferroni corrections for multiple comparisons when appropriate. We also report partial-eta squared (*ηp*^2^) and Cohen’s *d_av_* as measures of effect size. Question difficulty was manipulated *a posteriori*. Each participant answered 100 questions for each context. To ensure a similar amount of questions per difficulty level, we chose the cut-off points of 0.40 and 0.70. These limits divided all questions in each of the contexts in three levels with approximately one third of the questions per level.

### Accuracy and confidence: summary of results

Our main hypotheses pertained to the proportion of reported answers. However, differences in accuracy and confidence could affect the decision to report or withhold an answer. Thus, we first conducted analyses to check whether accuracy and confidence were similar between conditions. A full report of these analyses is presented in the Supplemental materials, but we included the main descriptive statistics in Table 2 for completeness. We also provide here a brief summary of these results. For accuracy, we found no differences per formality or answering strategy, and found the expected increase in accuracy as questions got easier. As per confidence, the same results were obtained but we also found a significant interaction between formality, answer strategy, and difficulty. Further analyses showed differences in confidence only for easy questions, and that metamemory measures such as calibration and resolution were not affected. However, confidence highly affects the decision to report an answer (Koriat and Goldsmith, 1996). In support of the strong association between confidence and report decisions, we found that the correlation between these measures ranged from 0.80 to 0.82 for the four contexts. To avoid any effect that differences in confidence could have on reporting decisions, we therefore included confidence as a covariate in the main analyses reported below. For completeness, we also report the same analyses without the covariate; notably, they produced highly similar results.

**Table 2:** Means (standard deviations) of the main measures per context and question difficulty.

	Formal		Informal	
	Constrained	Loose	Constrained	Loose
Accuracy				
Difficult	0.26 (0.07)	0.24 (0.11)	0.25 (0.07)	0.22 (0.11)
Intermediate	0.55 (0.14)	0.50 (0.14)	0.53 (0.14)	0.55 (0.16)
Easy	0.84 (0.12)	0.86 (0.10)	0.85 (0.09)	0.84 (0.12)
Confidence				
Difficult	47.25 (16.86)	49.27 (16.09)	48.95 (16.18)	49.08 (16.21)
Intermediate	55.54 (15.34)	54.70 (15.98)	53.97 (15.76)	56.33 (16.23)
Easy	73.62 (13.06)	77.79 (11.35)	76.64 (11.81)	75.57 (12.25)
Proportion reported (adjusted by the covariate)				
Difficult	0.66 (0.16)	0.68 (0.16)	0.68 (0.16)	0.73 (0.16)
Intermediate	0.65 (0.16)	0.68 (0.19)	0.71 (0.16)	0.69 (0.16)
Easy	0.69 (0.17)	0.68 (0.16)	0.70 (0.14)	0.68 (0.17)
Proportion reported (unadjusted by the covariate-raw proportion)				
Difficult	0.55 (0.21)	0.59 (0.19)	0.58 (0.20)	0.63 (0.21)
Intermediate	0.61 (0.19)	0.63 (0.22)	0.66 (0.20)	0.66 (0.19)
Easy	0.81 (0.12)	0.85 (0.11)	0.86 (0.10)	0.82 (0.13)
Report-criterion probability (*P_rc_*)				
Difficult	45.00 (20.05)	44.50 (19.32)	45.00 (20.06)	36.36 (16.96)
Intermediate	50.20 (16.82)	40.13 (23.23)	34.07 (18.65)	39.40 (18.56)
Easy	38.34 (18.04)	42.13 (18.25)	36.64 (21.85)	41.70 (22.90)

### Proportion of reported answers

We conducted an analysis of covariance (ANCOVA) with the three variables, formality, answering strategy, and difficulty, using the *ez* package (Lawrence, 2016) in R Core Team (2020). We entered the proportion of reported answers as dependent measure and confidence as covariate. Entering confidence as a covariate adjusted the reporting rate and eliminated any effect that was a consequence of prior differences in confidence. For example, the ANCOVA below equated confidence for the three levels of question difficulty. If question difficulty had an effect in the proportion of reported answer beyond its effect in confidence, then the ANCOVA should show differences in that variable.

In addition, entering confidence as a covariate also changed the meaning of our measure. As confidence was equated across all conditions, this measure is better interpreted as showing whether participants were conservative or liberal with respect to their own confidence level. Thus, the absolute values of the reporting rates adjusted by the covariate, reported in Table 2, have no direct interpretation and the interest is in the comparison between contexts and difficulty levels. Higher adjusted rates are interpreted as a liberal reporting policy (i.e., a focus in informativeness) and lower adjusted rates are interpreted as a conservative policy (i.e., a focus in accuracy).

The 3 × 2 × 2 ANCOVA showed a more liberal reporting strategy in the informal (*M* = 0.70, *SD* = 0.15) than in the formal contexts (*M* = 0.68, *SD* = 0.16), *F*(1, 24) = 18.56, *p* < 0.001, ηp^2^ = 0.44, and no differences by answering strategy, *F*(1, 24) = 2.40, *p* = 0.135, ηp^2^ = 0.09, or by question difficulty, *F*(2, 48) = 0.17, *p* = 0.844, ηp^2^ = 0.01. This latter result indicates that any effect of question difficulty on actual reporting rates was due to differences in confidence. Descriptive statistics of proportions adjusted by the covariate as well as unadjusted values are presented in Table 2. The interactions formality x difficulty, *F*(2, 48) = 3.33, *p* = 0.044, ηp^2^ = 0.12, and answering strategy x difficulty, *F*(2, 48) = 3.95, *p* = 0.026, ηp^2^ = 0.14, were both significant.

To ensure that pairwise comparisons were made over the response means adjusted by the confidence covariate and not over the raw, unadjusted, means, we used the *emmeans* package (Lenth, 2019) in R Core Team (2020). As our main interest was the reporting strategy depending on both social contexts (formality and answering strategy), we focused on pairwise comparisons within each difficulty level. For each interaction, six pairwise comparisons were taken into account with Bonferroni’s correction and alpha level set at 0.008. For difficult questions, participants applied a more liberal reporting policy in the informal (*M* = 0.70, *SD* = 0.16) than in the formal context (*M* = 0.67, *SD* = 0.15), *t*(71.9) = 2.98, *p* = 0.003, *d_av_* = 0.18, and also in the loose (*M* = 0.71, *SD* = 0.15) than in the constrained context (*M* = 0.67, *SD* = 0.16), *t*(71) = 2.97, *p* = 0.004, *d_av_* = 0.21. For the intermediate questions, participants also applied a more liberal policy in the informal (*M* = 0.70, *SD* = 0.16) than in the formal context (*M* = 0.67, *SD* = 0.17), *t*(71.9) = 3.93, *p* < 0.001, *d_av_* = 0.23, and there were no differences for easy questions.

Finally, we also conducted three analyses 2 formality × 2 answering strategy, one for each difficulty level, without the covariate to test the robustness of the previous analyses. The results mostly replicated the pattern above. For difficult questions, both formality and answer strategy influenced the proportion of answers reported, *F*(1, 24) = 4.90, *p* = 0.037, ηp^2^ = 0.17 and *F*(1, 24) = 7.11, *p* = 0.014, ηp^2^ = 0.23, respectively. Consistent with the more liberal criterion found in the analyses with the covariate, participants reported more answers in informal (*M* = 0.60, *SD* = 0.20) than in formal contexts (*M* = 0.57, *SD* = 0.19) and in loose (*M* = 0.61, *SD* = 0.19) than in constrained contexts (*M* = 0.56, *SD* = 0.20). For intermediate questions, participants also reported more answers in the informal (*M* = 0.66, *SD* = 0.18) than in the formal context (*M* = 0.62, *SD* = 0.20), *F*(1, 24) = 7.54, *p* = 0.011, ηp^2^ = 0.24. For easy questions, none of the main effects were significant, but the interaction was, *F*(1, 24) = 12.16, *p* = 0.002, ηp^2^ = 0.34. The interaction showed that participants reported more answers in the formal loose context than in the formal constrained, *t*(24) = 3.47, *p* = 0.002, *d_av_* = 0.35, but that they also reported more answers in the informal constrained than in the informal loose context, *t*(24) = 2.12, *p* = 0.044, *d_av_* = 0.30 (see Table 2 for descriptive statistics). As exploratory analyses with the covariate did not suggest a similar interaction, we will not discuss it further.

### *P_rc_* (report-criterion probability) analyses

Using the classical approach to data analysis, the above results showed that the incentives structure of social contexts only affected reporting strategy for intermediate and difficult items. To further verify statistically this conclusion that participants decided whether a response should be reported or withheld based on the confidence threshold, we employed the *P_rc_* analysis approach (see Introduction). As in the previous analyses, a higher *P_rc_* value means that fewer answers are reported and a more conservative strategy is employed, that is, the focus is on accuracy; the opposite is true for lower *P_rc_*. We computed *P_rc_* for each participant on each of the four social contexts. For completeness, we report *P_rc_* for each context and difficulty level in Table 2. Then, we examined the confidence distributions for the easy, intermediate, and difficult questions to identify the proportion of responses at each difficulty level that fall in the range between the highest and lowest *P_rc_* value for the four contexts. When the effect of social context is stronger for a given difficulty level, then there should be more responses falling between the highest and lowest *P_rc_*.

Following that logic, we computed the number of responses rated with confidence between the minimum and maximum *P_rc_* per participant and difficulty level and entered the results into a one-way ANOVA within-participants, using difficulty as a factor. The results showed significant differences, *F*(2, 48) = 24.72, *p* < 0.001, *η*^2^ = 0.51. Pairwise comparisons showed that the number of responses between the minimum and maximum *P_rc_* was higher for the difficult (*M* = 23.32, *SD* = 12.03, *CI* 18.6, 28.04]) and intermediate (*M* = 25.08, *SD* = 11.83, *CI* [20.44, 29.72]) questions than for the easy ones (*M* = 13.60, *SD* = 8.01, *CI* [10.46, 16.74]), *t*(24) = 4.49, *p* < 0.001, *d_av_* = 0.95 and *t*(24) = 6.34, *p* < 0.001, *d_av_* = 1.14, respectively, without differences between difficult and intermediate questions, *t*(24) = 1.54, *p* = 0.137, *d_av_* = 0.15. In sum, this analysis confirmed that the incentives structure of social contexts has a stronger effect in reporting strategies when answering intermediate and difficult questions than when answering easy questions.

## Discussion

In this study, we examined context-dependent conversational pragmatics from the perspective of the speaker. In particular, we investigated the effect of the incentive structure subjacent to different social contexts and levels of question difficulty on the amount of information the speakers share. Participants answered general-knowledge questions covering all levels of difficulty (easy, intermediate, difficult) and decided whether to report or withhold their answers in a particular social context. The main results showed that the incentive structure of social contexts, categorized as formal or informal or as constrained or loose, affected reporting rates and thus the amount of information speakers shared in these contexts. That is, the results confirmed our hypotheses regarding the influence of the formality of the context and, crucially, on the strategy that the context elicits. In particular, we expected that in the informal social contexts and those that elicit a loose pattern of answers, participants would favor informativeness and consequently report more information than in the other conditions. This shows that social context influences reporting strategies, thus confirming suggestions made by previous studies of conversational pragmatics (Ackerman and Goldsmith, 2008).

The results also showed that the influence of social context vanishes as the certainty regarding the answers’ correctness increases, in particular with easy questions. This supports our third hypothesis regarding the relationship between the level of difficulty of the questions and the incentives structure of social context. We hypothesized that if the question difficulty is more relevant than the social context’s incentives structure, then no differences in memory reporting across social contexts should be found. However, we did find differences for intermediate and difficult answers, supporting the idea that the incentives structure of the social contexts plays a bigger role in memory reporting for these more difficult questions as opposed to answering easy ones. These differences were found in all three types of analyses conducted: the adjusted ANOVA and *P_rc_* as well as the unadjusted analyses. That is, we analyzed participants’ reported answers separately from confidence in the unadjusted analyses and jointly in the adjusted ANOVA and *P_rc_,. P_rc_* was purposely designed to assess changes in the criterion of memory reporting based on the confidence ratings and thus allows to study threshold changes. Thus, *P_rc_* provides stronger evidence than the adjusted ANOVA on the relationship of the reporting strategies implement by participants, confirming the role of confidence in these decisions. In general, the results are highly consistent with the framework of the strategic regulation of memory reporting by Koriat and Goldsmith (1996), as extended by Goldsmith (2016): contextual factors and communication norms stemming from different contexts alter participants’ perception of the rewards and penalties involved in providing more or less information and, as a consequence, affect the amount of information they report.

This research expands and better characterizes the influence of social contexts on memory reporting. Previous research manipulated only one context dimension, formal vs. informal (Martín-Luengo et al., 2018, see text footnote 1), but here we also manipulated the general answering strategy expected in a given context and showed that there are no binary categories (such as ‘formality’) in which all the possible scenarios elicit the same incentives structure and the same reporting strategy. The focus on the incentives structure pertinent to different social contexts allowed us to further investigate other nuances of formal and informal contexts. Overall, the results show that different incentives for reporting or withholding answers are perfectly possible within the main category of formality proposed previously (Martín-Luengo et al., 2018). The results highlight the significant role of incentives for reporting depending on the social context and the variety that any broad category of context can include.

This research also showed that the strategic regulation of the informativeness-accuracy trade-off can be investigated with general-knowledge questions of all difficulties. Previous research in conversational pragmatics and memory reporting was conducted with difficult questions only (Martín-Luengo et al., 2018, see text footnote 1), following the logic that only when the questions are difficult there is a need to regulate the amount of information provided through reporting or withholding answers. However, the present data show that people also regulate the amount of provided information for easy and intermediate questions, as shown by the proportion of the reported answers. For easy and intermediate answers participants do not always report their choices, which indicates that participants need to regulate their memory reporting not only at the higher levels of difficulty but that they also do it with easy and intermediate questions.

However, we found that (1) for easy questions social context did not affect reporting, whereas (2) for intermediate questions only formality affected reporting, and, finally, (3) for difficult questions both types of context influenced reporting. These results show that the effect of social context on memory reporting is more complex than previously suggested. When questions are intermediate or difficult, threats to our self-image and the way people present themselves may be more evident, which could increase the perceived strength of the rewards and penalties for focusing on accuracy or informativeness in a given situation. However, for easy questions participants are more prone to ignore the context and its incentive structure, maybe because the chances of a mistake are low and the perceived consequences to self-image are also minimal.

We expected that social context may not affect reporting strategies for easy questions because most of them would be rated with high confidence and will thus be reported. However, a similar argument could be made for answers to difficult questions. If they are rated with low confidence, then they should be mostly withheld. From this perspective, only the condition with intermediate questions should be sensitive enough to show the effect of social context. However, this idea was not supported because the results showed an effect of both contexts for difficult questions. One explanation for this is that, as mentioned, the incentives and penalties for focusing on accuracy or informativeness may be perceived as being stronger when questions are difficult. Another explanation is that difficult questions were not difficult enough. Indeed, confidence for difficult questions was near 50%, which suggests that participants did not perceive questions as that much difficult. Arguing for the contrary, however, accuracy for difficult questions was only around 0.25, which is the baseline/chance level in a four-alternative test. If participants were in fact overconfident and considered difficult questions as intermediate, that could explain the effect of social context for these questions. Future research should try to test both of these alternatives to further advance our understanding of the effect of perceived incentives of social situations on memory reporting strategies.

Finally, another novel aspect of the present research is the use of multiple-choice task instead of cued-recall or free-recall questions. In previous studies, cued-recall questions were used as a proxy to what a conversation is, that is, a sequence of comments or questions and answers. This notwithstanding, when we are involved in a conversation, sometimes we also provide options along with our questions. Recognition tests are known to be easier than cued-recall tests because they are affected by familiarity (Tulving, 1985; Richardson-Klavehn and Bjork, 1988; Martín-Luengo et al., 2012), and at the same time they provide a straightforward way for experimental manipulations of individual items and easy quantification of results. In addition, our research shows that the effect of social context on memory reporting goes beyond the nature of the answers.

### Limitations

While showing novel findings, this study also has some limitations. First, the computerized administration of the questions is not particularly representative of how a natural conversation or interview unfolds. Previous research used different methods to try to mimic conversational exchanges. For example, Smith and Clark (1993) presented general knowledge questions orally (see also Kim, 2013, for a similar procedure). In the present case, due to the manipulation of the social contexts we decided against an oral experiment because it would have likely been more difficult for participants to imagine themselves in different social contexts represented by the very same experimenter than identifying the different social contexts using a graphical image. Second, context changed from one question to the next one in random order. While a block design with one block for each social context could have been more ecological, we decided to change contexts pseudorandomly as a way to test the strength of the implications of each context: a block design could have allowed participants to set a strategy a priory on each context-block, thus decreasing the variability of the results. Notably, in spite of the repetitiveness and the randomness in the design, the context manipulation was successful, which indeed suggests that the effect would have been stronger in a more natural situation with fewer repetitions. Future research should strive for more naturalistic experimental settings without compromising the quality of the results. We acknowledge that a conversation means more than answering questions, but in a more realistic conversational setting there would not have been much control over the manipulation of the variables of interest.

In line with these limitations, a more naturalistic procedure could have allowed to collect other conversational markers such as silences, hesitations, etc. These markers convey a large amount of valuable information that may help further understand participant’s conversational behavior. Future research on memory reporting would be greatly improved by the inclusion of such conversational markers in the context of a more naturalistic conversation.

The aforementioned limitations stem from the selection of a method with a restricted ecological validity. Yet, this particular paradigm and procedure were chosen to be able to isolate the influence of the specific experimental variables. This is particularly important in research lines that are not widely developed and for which it is necessary to set the grounds to build up knowledge.

Another important factor to consider is that each individual may react and behave in a particular social context in a rather varied way. Reactions can vary depending on the incentive structure. However, we did not directly manipulate incentives, which was instead done by proxy through the modeled social situations. One possibility to directly manipulate the incentive structure could have been to introduce monetary incentives, but that manipulation would have made the experimental set-up even further removed from a typical conversation situation. In addition, reactions can vary even depending on the emotional, psychological, or cognitive state of the same person. The present research was not intended to study such individual differences and that is why we framed the social contexts with the most prototypical and representative situations. Participants’ responses to prototypical situations inform us about what they perceive to be the incentive structure in these situations and what they think is the most appropriate reporting strategy. Thus, the results presented in this research are informative about general patterns of memory reporting, the perceived expectations in a given social context, and what the usual behavior in response to social challenges could be, and should not be interpreted as indicative of individual responses in particular situations. In line with this, further studies could also use this approach to study communicative behavior of different social and professional groups.

Finally, we want to stress that these results were found in a within-participant design; to the best of our knowledge, this procedure has not yet been tested in a between-participant design. There are metamemory phenomena that depend on this important feature. For example, font size only affects judgments of learning in within-participants designs (Chang and Brainerd, 2022). Thus, it might be that the results would have been different if a between-participants design was used. To test whether our findings are also design-dependent, further research should test the effects of the incentive structure on metamemory decisions in between-participant designs. Moreover, although we report two different convergent statistical analysis, future research could extend this approach and also include other types of analysis strategies, such as generalized linear mixed models.

## Concluding remarks

This study showed the importance of the incentives structure of a given social context for deciding on the best reporting strategy, the one focused on accuracy and reporting fewer answers or the one focused on informativeness and hence reporting more answers. In addition, this research also showed that the difficulty of the materials is relevant for understanding reporting strategies, with easy questions being seemingly less sensitive to variations in social context. This helps advance our understanding of how social situations shape and affect communication flow, in particular how much information people are willing to share. Future research should study other ways people can use to modify their discourse behavior to further reveal the underlying principles guiding human communication.

## Data availability statement

The raw data supporting the conclusions of this article will be made available by the authors, without undue reservation.

## Ethics statement

The studies involving human participants were reviewed and approved by ethics committee of the National Research University Higher School of Economics, Moscow. The patients/participants provided their written informed consent to participate in this study.

## Author contributions

BML and YS acquired funding for research. BML conducted the data collection and completed the initial writing. BML and KL analyzed the data collection. All authors conducted the conceptualization of the research study, and revised and contributed to the final version.

## Funding

This article is an output of a research project implemented as part of the Basic Research Program at the National Research University Higher School of Economics (HSE University) and was carried out using HSE Automated system of non-invasive brain stimulation with the possibility of synchronous registration of brain activity and registration of eye movements.

## Conflict of interest

The authors declare that the research was conducted in the absence of any commercial or financial relationships that could be construed as a potential conflict of interest.

## Publisher’s note

All claims expressed in this article are solely those of the authors and do not necessarily represent those of their affiliated organizations, or those of the publisher, the editors and the reviewers. Any product that may be evaluated in this article, or claim that may be made by its manufacturer, is not guaranteed or endorsed by the publisher.

## Supplementary material

The Supplementary material for this article can be found online at: https://www.frontiersin.org/articles/10.3389/fpsyg.2023.1004524/full#supplementary-material

## Footnotes

^1^Martín-Luengo, B., Nunez Vorobiova, A., Feurra, M., Myachykov, A., and Shtyrov, Y. (2023). The role of left middle frontal gyrus in context-dependent communication under pressure: rTMS evidence. [Manuscript under review].
