# Supplementary material for: Conversational pragmatics: memory reporting strategies in different social contexts
Source: Front Psychol. 2023 May 25;14:1004524. doi: 10.3389/fpsyg.2023.1004524 (PMC10249957; doi:10.3389/fpsyg.2023.1004524)
Supplement: Supplementary file 1 [file Table_1.DOC]

**Conversational Pragmatics: Memory Reporting Strategies in Different Social Contexts**

**Supplemental Materials**

In the research reported in the main manuscript, we were interested in the reporting strategies depending of the incentive structure of different social contexts. In order to properly examine reporting strategies, we first needed to verify that the accuracy and the confidence levels were equally distributed among conditions. Thus, we report here analyses of accuracy and confidence per context and difficulty level.

**Accuracy**

See Table S1 for descriptive statistics. There were no differences for formality, *F*(1, 24) = 0.07, *p* = .789, η_p_² < .01, or answering strategy, *F*(1, 24) = 1.72, *p* = .202, η_p_² = .07, and there was the expected effect of question difficulty, *F*(2, 48) = 541.83, *p* < .001, η_p_² = .96. No interactions were significant.

**Table S1.** Means (Standard Deviations) of Accuracy and Confidence per Context and Question Difficulty.

|  | FORMAL | | INFORMAL | |
| --- | --- | --- | --- | --- |
|  | Constrained | Loose | Constrained | Loose |
| Accuracy |  |  |  |  |
| Difficult | .26 (.07) | .24 (.11) | .25 (.07) | .22 (.11) |
| Intermediate | .55 (.14) | .50 (.14) | .53 (.14) | .55 (.16) |
| Easy | .84 (.12) | .86 (.10) | .85 (.09) | .84 (.12) |
| Confidence |  |  |  |  |
| Difficult | 47.25 (16.86) | 49.27 (16.09) | 48.95 (16.18) | 49.08 (16.21) |
| Intermediate | 55.54 (15.34) | 54.70 (15.98) | 53.97 (15.76) | 56.33 (16.23) |
| Easy | 73.62 (13.06) | 77.79 (11.35) | 76.64 (11.81) | 75.57 (12.25) |

Note: data in this table are also presented in the main text. We repeat the information for the convenience of the reader.

**Confidence**

See Table S1 for descriptive statistics. Confidence was rated after the answer was provided but before the context was presented. Thus, we expected an effect of difficulty, with higher confidence as question difficulty decreased, and no differences by context. An ANOVA with confidence in the answers showed no differences for formality, *F*(1, 24) = 0.40, *p* = .534, η_p_² = .02, differences for answering strategy, *F*(1, 24) = 4.77, *p* = .039, η_p_² = .17, and an effect of question difficulty, *F*(2, 48) = 152.22, *p* < .001, η_p_² = .86. There was also an interaction between the three variables that qualified the main effects, *F*(2, 48) = 5.64, *p* = .006, η_p_² = .19. There were no other interactions.

Exploration of the interaction showed that differences between contexts were limited to the easy questions. Easy questions that were placed later in the formal-loose context were rated with higher confidence than those placed later in the formal-constrained context, *t*(24) = 4.09, *p* < .001, *d* = 0.33, with no other differences regarding contexts. This difference was unexpected because, as mentioned, contexts were presented after confidence was registered and we fully counterbalanced conditions.

**Calibration**

To further test whether differences in confidence depending on the type of context affected metamemory, we also analysed confidence in relation with accuracy through calibration and resolution analyses (see Table S2 for the main descriptive statistics). To ensure enough data points in each condition, we collapsed the 11 confidence points into five. Confidence levels 0 and 100 are qualitatively different than others because they show that the participant was guessing or completely certain, while the intermediate levels show different degrees of uncertainty. Thus, we collapsed levels 10-20-30, 40-50-60, 70-80-90, and maintained levels 0 and 100.

**Table S2.** Means (Standard Deviations) of the Calibration Index and Gamma

|  | FORMAL | | INFORMAL | |
| --- | --- | --- | --- | --- |
|  | Constrained | Loose | Constrained | Loose |
| Calibration Index |  |  |  |  |
| Difficult | 0.18 (0.10) | 0.20 (0.10) | 0.18 (0.12) | 0.21 (0.12) |
| Intermediate | 0.11 (0.07) | 0.10 (0.07) | 0.09 (0.06) | 0.11 (0.05) |
| Easy | 0.07 (0.06) | 0.07 (0.05) | 0.06 (0.06) | 0.07 (0.06) |
| Gamma Correlation |  |  |  |  |
| Difficult | .12 (.49) | -.06 (.47) | .15 (.50) | -.03 (.41) |
| Intermediate | .30 (.30) | .29 (.33) | .40 (.27) | .41 (.30) |
| Easy | .63 (.36) | .62 (.33) | .58 (.45) | .60 (.34) |

Calibration measures the extent to which confidence matches accuracy. A participant would be perfectly calibrated when their answers rated with 50% confidence are 50% accurate and so on. A calibration index (see Brewer et al., 2002 for the calculations) of 0 indicates a perfect calibration and higher values indicate worse calibration. There were no differences in formality, *F*(1, 24) = 0.22, *p* = .642, η_p_² < .01, or answering strategy, *F*(1, 24) = 3.62, *p* = .069, η_p_² = .13. Calibration was better as difficulty decreased, *F*(1, 24) = 27.46, *p* < .001, η_p_² = .53, which is consistent with the idea that good knowledge is accompanied by good knowledge about knowing. No interaction was significant.

**Resolution**

Similarly, differences in confidence did not affect resolution. Resolution measures the degree to which confidence can discriminate between correct and incorrect responses and is measured with the Goodman and Kruskal gamma correlation. Gamma ranges from +1, perfect positive discrimination (i.e., all correct answers are rated with the highest confidence), to -1, meaning perfect negative discrimination (i.e., all the correct responses are rated with the lowest confidence). A gamma equal to zero is interpreted as that participants’ confidence does not discriminate between correct and incorrect responses. Gamma was computed for each individual and condition and pooled together. There were no differences in formality, *F*(1, 24) = 0.56, *p* = .461, η_p_² = .023, or answering strategy, *F*(1, 24) = 2.72, *p* = .113, η_p_² = .10, and resolution improved as difficulty decreased, *F*(1, 24) = 39.93, *p* < .001, η_p_² = .62. There were no interactions.

Taken together, the results indicate that the highest confidence in answers that were later in the formal-loose condition for easy questions did not affect other metamemory measures. However, reporting or withholding an answer is highly based on confidence and that result could explain differences in the proportion of reported answers. To control for this alternative explanation, in the analyses of the proportion of reported answers in the main text we included confidence as a covariate.

**References**

Brewer, N., Keast, A., & Rishworth, A. (2002). The confidence–accuracy relationship in eyewitness identification: The effects of reflection and disconfirmation on correlation and calibration. *Journal of Experimental Psychology: Applied, 8*, 44–56. https://doi.org/10.1037/1076-898X.8.1.44
